# Retraction: Colonic Angiolipoma: An Extremely Rare Tumor Clinically Masquerading as Acute Appendicitis

**DOI:** 10.7759/cureus.r120

**Published:** 2024-01-25

**Authors:** Abdullah S Alfaraj, Asma A Almohamad, Nader S Alqabbani, Mohammed H Alghazwi, Abdulkarim M Alharbi, Amina A Ahmed, Marwan K Aljumah, Naif Y Aljumaah, Hawra E Kadhem, Hassan H Al Jalooud, Zahra S AlMohsen, Kawkab M Alharbi, Hassan E Al Abbas, Fatimah B Al Khalifah, Faisal Al-Hawaj

**Affiliations:** 1 General Practice, Al Naeriyah Hospital, Naeriyah, SAU; 2 College of Medicine, Ibn Sina National College for Medical Studies, Jeddah, SAU; 3 College of Medicine, Arabian Gulf University, Manama, BHR; 4 College of Medicine, Prince Sattam Bin Abdulaziz University, Al-Kharj, SAU; 5 College of Medicine, King Saud University, Riyadh, SAU; 6 College of Medicine, Xi'an Jiaotong University, Xi'an, CHN; 7 College of Medicine, Qassim University, Qassim, SAU; 8 College of Medicine, Vision College, Riyadh, SAU; 9 College of Medicine, King Faisal University, Hofuf, SAU; 10 College of Medicine, Princess Nourah Bint Abdulrahman University, Riyadh, SAU; 11 College of Medicine, Imam Abdulrahman Bin Faisal University, Dammam, SAU

The Editors-in-Chief have retracted this article. Concerns were raised regarding the identity of the authors on this article. Specifically, Faisal Alhaway and Malak Shammari have stated that they were added to this article without their knowledge or approval. The identity of the other authors could also not be verified. As the appropriate authorship of this work cannot be established, the Editors-in-Chief no longer have confidence in the results and conclusions of this article.

